# Changes in the sediment microbial community structure of coastal and inland sinkholes of a karst ecosystem from the Yucatan peninsula

**DOI:** 10.1038/s41598-022-05135-9

**Published:** 2022-01-21

**Authors:** Pablo Suárez-Moo, Claudia A. Remes-Rodríguez, Norma A. Márquez-Velázquez, Luisa I. Falcón, José Q. García-Maldonado, Alejandra Prieto-Davó

**Affiliations:** 1 Unidad de Química-Sisal, Facultad de Química, Universidad Nacional Autónoma de México, 97356 Sisal, Yucatán Mexico; 2 Posgrado en Ciencias del Mar y Limnología, Unidad de Química-Sisal, Universidad Nacional Autónoma de México, 97356 Sisal, Yucatán Mexico; 3 Laboratorio de Ecología Bacteriana, Instituto de Ecología, Universidad Nacional Autónoma de México, 97302 Sierra Papacal, Yucatán Mexico; 4 Centro de Investigación y de Estudios Avanzados del Instituto Politécnico, Antigua Carretera a Progreso Km. 6, 97310 Mérida, Yucatán Mexico

**Keywords:** Microbiology, Ecology

## Abstract

The karst underground river ecosystem of Yucatan peninsula is composed of cave systems and sinkholes. The microbial diversity of water from this underground river has been studied, but, structure of the microbial community in its cave sediments remained largely unknown. Here we describe how the microbial community structure of these sediments changes due to different environmental conditions found in sediment zones along the caves of a coastal and an inland sinkhole. We found that dominant microbial groups varied according to the type of sinkhole (Coastal: Chloroflexi and Crenarchaeota; inland: Methylomirabilota and Acidobacteriota) and that the community structures differed both among sinkhole types, and within the sediment zones that were studied. These microorganisms are associated with different types of metabolism, and differed from a microbial community dominated by sulfate reducers at the coastal sinkhole, to one dominated by methylotrophs at the inland sinkhole, suggesting there are biogeochemical processes in the coastal and inland sinkholes that lead to changes in the microbial composition of the underground river ecosystem’s sediments. Our results suggest sediments from unexplored sinkhole caves are unique environmental niches with distinct microbial assemblages that putatively play an important role in the biogeochemical cycles of these ecosystems.

## Introduction

The Yucatan peninsula is a limestone platform with an underground river ecosystem that is considered to be one of the most extensive active karst carbonate systems in the world^1,2^. In this underground ecosystem there are sinkholes, that are locally referred to as “cenotes” (from the Mayan word *ts'onot)*, where the cave ceiling has partially or completely collapsed, due to the dissolution of the karst carbonate rock that forms it^3^. These cenotes are thus exposed to the atmosphere above the ground.

Sinkholes are located on the Yucatan peninsula ranging from the central region to coastal areas, with wide morphological, physicochemical, and biological variations associated with rainfall seasonality, which also affects the vertical stratification in sinkhole water columns^3–6^. In the central region of the peninsula, most of these inland sinkholes are exclusively freshwater systems and, in contrast, those close to the coast have a marked vertical stratification of surface freshwater and deeper saltwater^3,7^. Differences in physicochemical variables between sinkholes have been correlated with variation in ichthyofauna^5^, and bat populations^8^, in addition to other organisms. For example, the ichthyofauna and invertebrate diversity of the coastal sinkholes are more similar to the marine environment than that found at inland sinkholes^3,9^.

The microbial communities found in the karst cave systems around the world have been associated with important roles in biogeochemical cycles^10,11^, alternative primary production^12^, trophic interactions that impact food webs^13^, as indicators of the health of the ecosystem^6,14^, and as a source of new products of biotechnological importance^15^. However, despite the potential essential roles of microorganisms in the Yucatan groundwater ecosystem, most studies regarding these cave systems have focused on cave morphology and hydrology^1,3^, macrofaunal diversity^8,16^, and pollution^14,17,18^. The microbial communities associated with these unique environments remain largely unexplored. The few studies describing microbial diversity in the cave system environment of the Yucatan peninsula focused on the microbial communities in speleothems and water from the sinkholes^2,6,13^, while the sediment microbial diversity has never been thoroughly described. A study of the El Zapote coastal sinkhole reported a higher bacterial diversity at the speleothems’ surface when compared with that of sinkhole water of the same depth^2^. The authors reported a dominance of select microbial groups associated with metabolic functions that included sulfur-oxidizing autotrophs, nitrite oxidizers, and anaerobic ammonium oxidizers^2^. Other previous studies reported highly diverse microbial communities in karst sinkhole waters and a drinking water well from Yucatan groundwater characterized by warm temperatures and high nutrient input from human activity^6^. Brankovits et al.^13^ compared the microbial diversity across water masses from a sinkhole in the karst underground river ecosystem of the Yucatan peninsula. They found differences in the relative abundance of microbial functional groups (MFGs) associated with the type of water (freshwater vs saline) in the cave systems, and demonstrated the importance of microbial community composition in the carbon cycle and as a facilitator within the sinkhole trophic chain^13^.

In the current study, we describe and compare the microbial composition (Bacteria and Archaea) from three sediment zones in coastal and inland sinkholes. We describe how environmental characteristics found in these ecosystems are linked to differences in microbial community composition and putatively influence the roles microbes play in the main biogeochemical cycles within these sediments. To our knowledge, this is the first thorough description of microbial community structure in sinkhole sediment environments and their potential role in biogeochemical cycles within the underground river ecosystem. This study highlights the importance of continuous studies to reveal the microbial ecology of these sediments and better understand community roles in biogeochemical cycles within the Yucatan karst aquifer.

## Results

### Physical and chemical properties of water and sediments

The in situ variables of the water column and the sediment from sampling sites and zones are shown in Supplementary Fig. 1 and Table S1. Variability of the physicochemical parameters within the water column of the coastal sinkhole was observed along the three defined sediment zones (p < 0.001) (with an average of six samples per sediment zone at the coastal sinkhole and one measurement per zone at the inland), (Supplementary Fig. 1 and Table S2). Differences in the water column include higher salinity, temperature, conductivity, and Total Dissolved Solids (TDS) at the coastal sinkhole (Supplementary Fig. 1), with the water mirror (WM) samples showing the greater differences (Supplementary Fig. 1). Dissolved oxygen (DO) showed lower vertical variation concentrations in the coastal sinkhole (Supplementary Fig. 1).

A principal component analysis based on the environmental variables from water and sediment for the coastal and inland sinkholes emphasized the differences observed among the three sampled zones (Supplementary Fig. 2). Within both sinkholes, cavern and cave samples showed higher similarities in their physical and chemical variables, both in the water column and sediments (Supplementary Fig. 2 and Table S1), suggesting a unique environment in the cave system that differs from the WM in contact with the atmosphere. Furthermore, the physical and chemical analyses of the sediment samples showed higher concentrations of organic and inorganic carbon and total nitrogen at the coastal sinkhole, suggesting that saline intrusion allows for the input of marine organic and inorganic matter at this location. Further sampling with more replicates at both locations could help to confirm these observations.

### Microbial community structure from coastal and inland sinkholes

**Figure 1:**
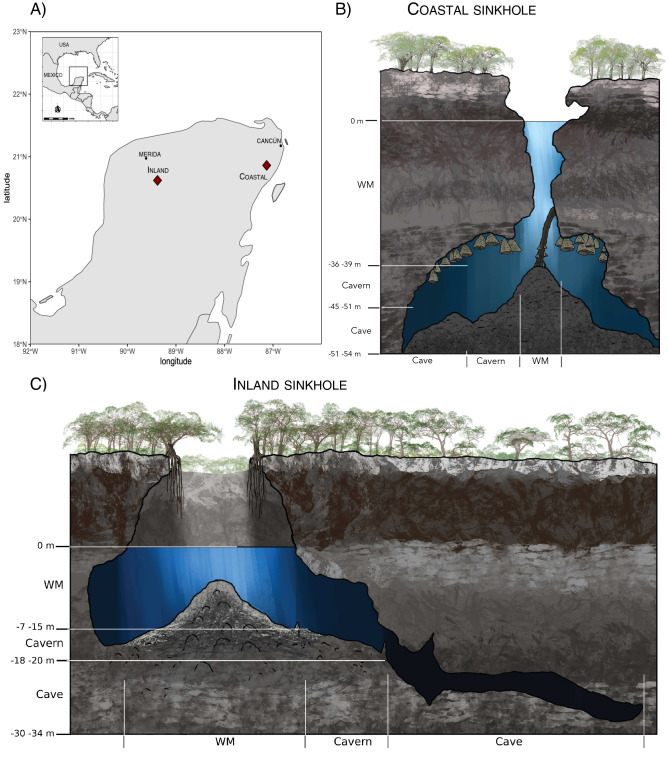
Study sites and sediment zones in the coastal and inland sinkholes from the Yucatan Peninsula. Each site is indicated by a diamond symbol (**A**), and the three sediment zones are indicated in the cartoons of the coastal (**B**) and inland sinkholes (**C**). Artwork based on divers’ descriptions drawn by Alberto Guerra.

A total of 14,227,212 reads were obtained from sequencing 104 sediment samples from the three zones in the coastal and inland sinkholes (Fig. 1). Following quality filtering processes 7,225,233 reads were analyzed, and 9422 amplicon sequence variants (ASVs) were found (Supplementary Table S3). Numbers of ASVs for each sediment zone in normalized and non-normalized data were similar for the three data groups (see “Methodology”), with the most diverse samples identified in the cavern and WM samples from both sinkholes (Supplementary Table S3). For the sediment samples, the number of sequences obtained ranged between 8204 and 210,852 per sample, while ASVs ranged between 246 and 2478 per sample. The median for reads for sediment samples was 48,557.5, while the median for ASVs was and 915 (Supplementary Table S4). Soil samples were associated with a lower level of microbial diversity than expected (Supplementary Table S4), with sequences ranging between 23,561 and 67,304 and ASVs between 351 and 480. The median reads and ASVs per soil sample were 43,351 and 415, respectively (Supplementary Table S4).

To analyze the microbial community structure of sinkhole sediments, three types of normalized ASV data analyses were performed (Supplementary Table S3). Rarefaction curves of the “observed ASVs” demonstrated that the number of ASVs reached a plateau, indicating adequate sampling efforts for the three data groups (Supplementary Fig. 3). From the normalized ASV data group 1 (110 samples and normalized count = 8204 reads) 9402 ASVs were obtained and classified to the most specific taxonomic level possible and corresponded to 61 phyla, 108 classes, 194 orders, 245 families, and 314 genera. A total of 1763 ASVs and 27% of total reads were identified as Archaea.

**Figure 2:**
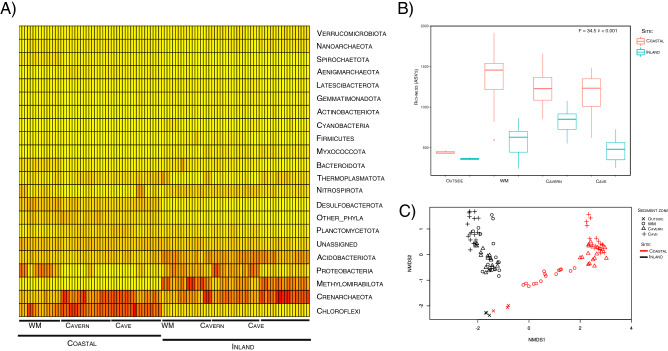
Differences in the sediment microbial communities from the coastal and inland sinkholes (data group 1). (**A**) Heat map of the 20 most abundant phyla. Columns represent sediment samples, rows depict phyla. Low abundance (yellow), high abundance (red). (**B**) Richness of ASVs within the sites and sediment zones from sinkholes. (**C**) Beta diversity analysis of the sediment samples. Non-metric multidimensional scaling (nMDS) plot based on the Bray–Curtis distance of 9402 ASVs (Figures created with Rstudio).

The environmental differences between the coastal and inland sinkholes (Supplementary Figs. 1 and 2; Table S1) related to variations in the microbial community structure from the sediments from the two distinct sinkholes. Taxonomic diversity analyses of the sediment samples in both sinkholes, at the phylum level, showed differences in their most abundant phyla (Fig. 2A). The sediment zones from the coastal sinkhole showed high abundances of Chloroflexi, Crenarchaeota, and Desulfobacterota, while that of the inland sinkhole had higher abundances of Crenarchaeota, Methylomirabilota, and Acidobacteriota (Fig. 2A). There was significantly higher richness in the coastal sinkhole than in the inland sinkhole (Fig. 2B). Non-metric multidimensional scaling analysis (NMDS) confirmed that the site characteristics determined the microbial community clustering which was corroborated by permutational multivariate analysis of variance (PERMANOVA) (F = 4.9, p < 0.001) (Fig. 2C). These results suggest that sinkhole sediment samples have microbial communities shaped by environmental characteristics unique to each type of sinkhole (coastal or inland), and that are distinct from that of the soil outside the cenote.

### Microbial community structure of sediment zones

**Figure 3:**
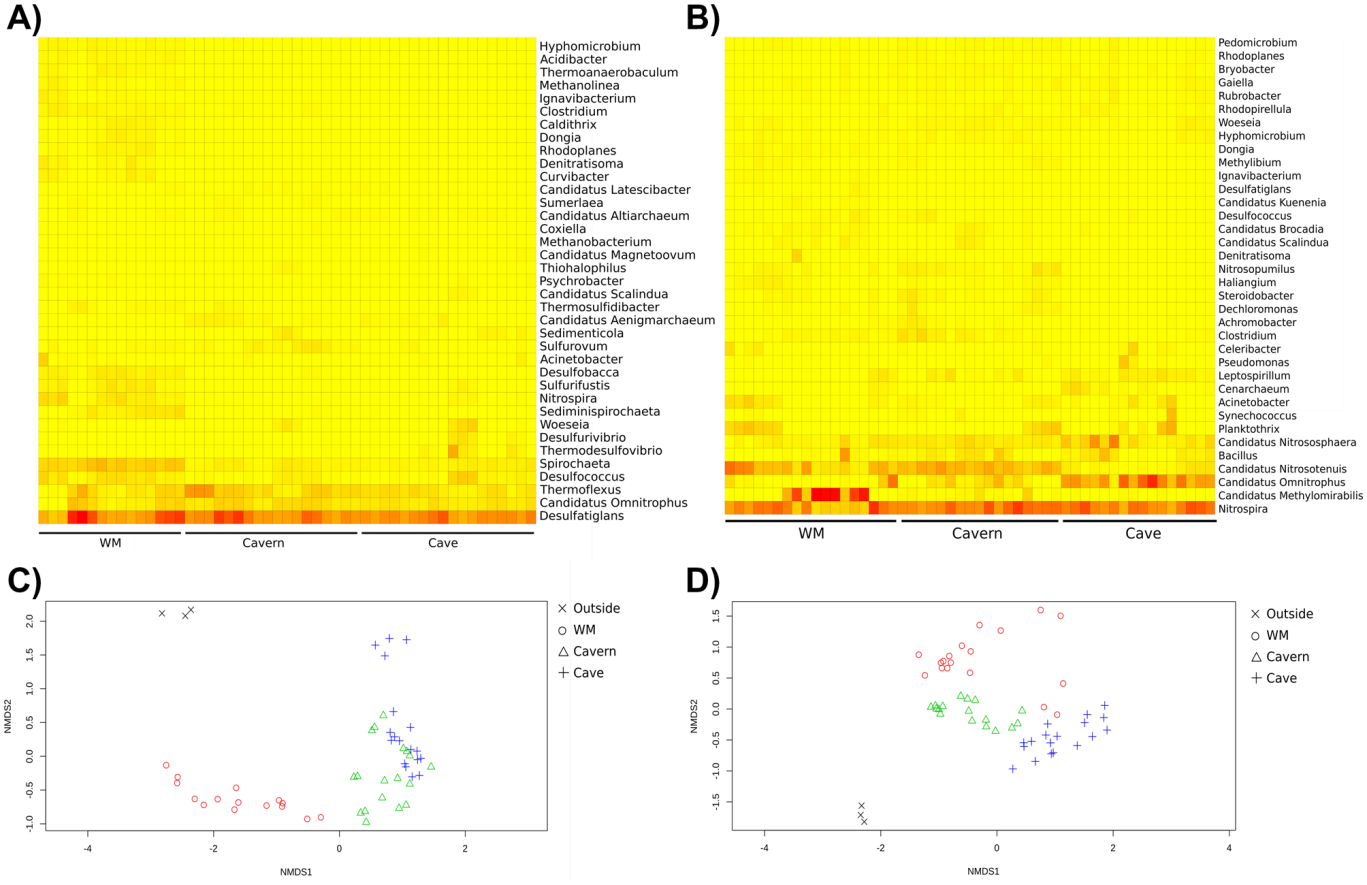
Taxonomic and beta diversity in the sediment zones. (1) Heat map of the 20 most abundant genera for each sediment zone in the coastal (**A**) and inland (**B**) sinkholes, the columns represent sediment sample, rows depict genera. Low abundance (yellow), medium abundance (orange) and high abundance (red). (2) Beta diversity analysis of the sediment samples. Non-metric multidimensional scaling (nMDS) plot based on the Bray–Curtis distance of 21,432 (**C**, Coastal sinkhole) and 8204 (**D**, Inland sinkhole) reads per sample.

For the coastal sinkhole dataset, 51 samples were normalized to a 21,432 read count based on the number of sequences from the library with the lowest sequencing depth within data group 2 (see “Methodology”). The most abundant families (i.e., highest percentage of total reads) were: Desulfatiglandaceae (5.2%), Anaerolineaceae (2.4%), Spirochaetaceae (1.6%), Thermoflexaceae (1.4%), Omnitrophaceae (1.1%) (Supplementary Fig. 4). A total of 254 genera were identified (Supplementary Fig. 4). The five most abundant genera in the 51 sediment samples included *Desulfatiglans* (5.2%), *Thermoflexus* (1.4%), *Candidatus Omnitrophus* (1.1%), *Spirochaeta* (1.0%), *Desulfococcus* (0.6%) (Fig. 3A and Supplementary Fig. 4). The inland sinkhole had 53 samples that were normalized to a 8204 read count based on the number of sequences from the library with the lowest sequencing depth within data group 3 (see “Methodology”). The most abundant families were Methylomirabilaceae (10.7%), Nitrosopumilaceae (7.8%), Nitrospiraceae (4.7%), Nitrosotaleaceae (2.2%), Vicinamibacteraceae (2.1%) (Supplementary Fig. 4). A total of 221 genera were identified (Supplementary Fig. 4). The five most abundant genera in the 55 sediment samples included *Nitrospira* (4.7%), *Candidatus Methylomirabilis* (2.8%), *Candidatus Nitrosotenuis* (2.0%), *Candidatus Omnitrophus* (1.4%), *Candidatus Nitrososphaera* (1.0%) (Fig. 3B and Supplementary Fig. 4).

A heatmap analysis of the 20 most abundant genera within the coastal sinkhole (data group 2) revealed that sediments of the cavern and cave showed similar abundances of some genera including *Desulfatiglans*, and *Thermoflexus*, while the WM at the coastal sinkhole had *Spirochaeta*, *Desulfobacca*, *Sulfurifustis*, *Nitrospira*, and *Sediminispirochaeta* as the most abundant genera (Fig. 3A). On the other hand, the inland sinkhole showed slight differences in the abundance of genera within sediment zones (data group 3). Genera including *Nitrospira*, and *Candidatus Nitrososphaera* were abundant in the cavern and cave. *Candidatus Omnitrophus* was most abundant in the cave and *Candidatus Nitrisostenuis* in WM, and in the cavern (Fig. 3B).

The highest richness of observed ASVs was found both in the cavern and WM, while the cave showed lower richness at both sinkholes (Supplementary Fig. 5). A high number of ASVs and genera were exclusive to each of the three sediment zones in the coastal (36.6% ASVs and 36% genera) and inland (23.2% ASVs and 15.7% genera) sinkholes. In the coastal sinkhole, of the 4007 ASVs and 203 genera found in the WM, 1886 ASVs (27.3%) and 78 genera (34.2%) were exclusive to this sediment zone, while 1935 ASVs (27.9%) and 97 genera (42.7%) were shared with the cavern and 1010 ASVs (14.7%) and 67 genera (29.4%) were shared with the cave. The cavern and cave shared 3230 ASVs (46.7%) and 82 genera (35.96%) (Supplementary Fig. 5). At the inland sinkhole, of the 2498 ASVs and 168 genera found in the WM, 344 ASVs (9.5%) and 12 genera (5.9%) were exclusive to this sediment zone, while 1985 ASVs (54.6%) and 151 genera (74%) in this zone were also observed at the cavern and 1002 ASVs (27.6%) and 78 genera (38.2%) were also observed at the cave. The cavern and cave shared 1177 ASVs (32.4%) and 81 genera (39.7%) (Supplementary Fig. 5). When comparing the soil samples from outside the sinkholes with the sediments form the different sinkholes zones, we found that a high percentage of ASVs and genera were exclusive to the outside control samples with 337 ASVs (63.3%) from the soil at the coastal sinkhole and 253 ASVs (56%) from the soil at the inland sinkhole. This suggests that the microbial communities from the sinkhole’s sediment are not influenced by those of the surrounding environment outside the sinkhole.

Furthermore, non-metric multidimensional scaling analysis (NMDS) with Bray–Curtis and weighted Unifrac distances, and a permutational multivariate analysis of variance (PERMANOVA) (F = 4.9, p < 0.001) showed that microbial communities clustered according to sediment zones in both sinkholes (Fig. 3C,D and Supplementary Fig. S6). Cavern and cave samples showed a tighter clustering than the WM samples did. Only three of 18 samples from the WM at the inland sinkhole grouped closer to the cavern and cave samples, however, these samples were closer in distance to the cavern itself (Fig. 3C,D and Supplementary Fig. S6).

### The potential metabolic function of the microbial communities

**Figure 4:**
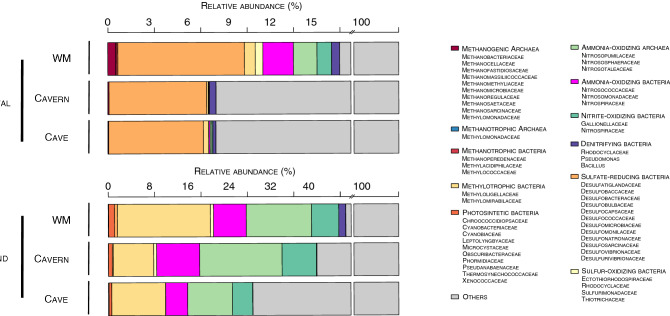
Relative abundance of the functional groups in the three sediment zones from coastal and inland sinkholes. WM, water mirror.

A review of literature on the potential metabolic function of the bacterial families from the sinkholes suggested that at least eleven main microbial functional groups (MFG) present at the coastal and inland sinkholes, with differences in their relative abundance according to the type of sinkhole (Fig. 4). This analysis suggested that the coastal sinkhole was dominated by sulfate-reducing bacteria, with highest abundance in the WM zone (8.1%), followed by the cavern and cave (6.1% for both zones). Other MFG identified in the WM zone included ammonia-oxidizing bacteria (1.9%) and ammonia-oxidizing archaea (1.5%), where other archaeal functional groups were observed. Methanogenic archaea were most abundant in WM (0.5%), followed by the cavern and cave (0.1% for both zones) (Fig. 4). The inland sinkhole was dominated by methylotrophic and nitrifying bacteria (the latter associated with ammonia and nitrite-oxidation). The WM zone of the inland sinkhole was dominated by methylotrophic bacteria (15.9%), ammonia-oxidizing archaea (AOA, 11.2%), ammonia-oxidizing bacteria (AOB, 5.6%), and nitrite-oxidizing bacteria (NOB, 4.7%). The cavern zone was dominated by AOA (14.1%), AOB (7.4%), methylotrophic bacteria (6.9%), NOB (5.9%), while the cave zone was dominated by methylotrophic bacteria (9.2%), AOA (7.7%) and, AOB (3.8%). The photosynthetic bacteria in the inland sinkhole were most abundant in the WM zone (1%) compared with the cavern (0.7%) and cave (0.5%). This MFG was absent in the cavern and cave from the coastal sinkhole (Fig. 4). These analyses suggest that the environmental characteristics of each sinkhole and their sediment zones may be responsible for the differences observed in the MFGs, highlighting the impact these microbes may have on major biogeochemical cycles in the sediments from the underground aquifer of the Yucatan peninsula.

### Comparative taxonomic profiling of sediments from several environments

**Figure 5:**
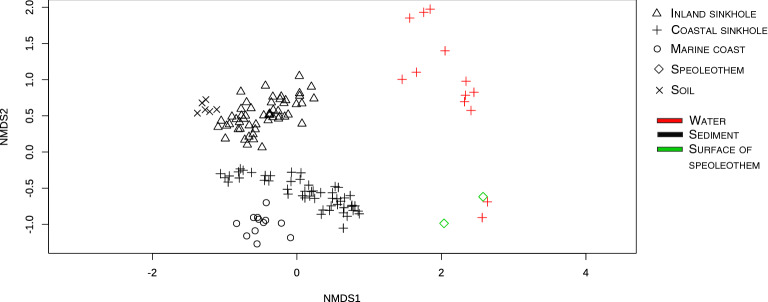
Beta diversity analysis of microbial communities from sediment, water and surface of speleothems from studies performed in the Yucatan peninsula (normalization of 3490 reads by sample).

To determine whether the sediment microbial community from sinkholes has a particular structure, we compared the microbial diversity dataset generated in this study (at the family level) against sequencing data from other sediments, water and speleothem samples from the Yucatan peninsula (Supplementary Table S5). In this comparative reanalysis, we found that the microbial community structure from sediment sinkholes clustered closer to marine sediments and soil than to water and speleothem samples from coastal sinkholes (Stinnesbeck’s work was performed in Zapote sinkhole^2^) (Fig. 5). Analysis of the taxonomic diversity (366 total families) from the different environments revealed sinkhole sediments (coastal and inland) were comprised 101 exclusive families, (i.e., families not shared with any other environment), 138 families shared with marine sediments, and 46 families shared with sinkhole water samples (Supplementary Fig. 7). These results highlight how sinkholes represent environments with microbial assemblages that could be influenced by nearby marine sediments but still possess a set of unique microbes. Differences between free-living water column bacteria and sediment associated bacteria inside the sinkholes suggest a shift in the metabolic potential of microbial communities in both niches and suggest they have a role in the biogeochemistry of these groundwater ecosystems.

## Discussion

Our results show that differences in environmental conditions between inland and coastal sinkholes, caused mainly by the inflow of seawater in the latter, influence the microbial community structure of their sediments. Furthermore, the microbial community structure also varied within the sinkholes and according to the sediment zone sampled, suggesting that a connection between the atmosphere in the outermost location of the sediments and sunlight creates an environment distinct from that found in deeper caves. Together with the different environmental factors that were measured (in situ physicochemical composition of water and sediment) these characteristics could drive niche-specific microbial community structures associated with the sediment zones. Additionally, beta-diversity analysis showed separate clustering of the sediment microbial communities from the coastal and inland sinkholes, and of the WM zone from cavern and cave zones at both sinkholes. Microbial community structure associated with karst environments have shown to be significantly influenced by environmental factors as seen in the Bahamian blue holes^19^, a coastal sinkhole^13^, a Floridan anchialine sinkhole^20^, and sediments from Chinese karst caves^21^.

Microbial communities from karst sediments can be limited by nutrients such as carbon, phosphorus, and nitrogen^21^, therefore, influencing their structure. Differences in the microbial community composition associated with multiple environmental factors (moisture, type of niche, nitrogen) were also reported in karst cave sediments from China^21^. Previous studies had shown there was no effect on the alpha diversity of water column assemblages in the Yucatan groundwater associated with the type of sinkhole (inland or coastal)^6^. However, this observation may be limited due to the low sample number used in the study^6^. For other karst sinkholes, the microbial community dynamics differ between the water column and the sediments^6,22–24^.

The karst caves and sinkholes of the underground river in Yucatan are characterized by low phosphorus concentrations and high levels of nitrate, mostly related to Anthropocentric activities (urban developments, farms and agriculture)^3^. The inland sinkhole at Noh Mozón showed the highest concentration of nitrate detected in the study and, not surprisingly, the area is surrounded by agricultural fields. The presence of organic matter in the sinkholes from the Yucatan peninsula are highly dependent on the connection between the cave systems, on the levels of exposure to light, and on their morphology^3^. High concentrations of organic carbon (661 ± 132 μM) and methane (6466 ± 659 nM) have been reported in the top layer of the water masses in coastal sinkholes before^13^. In this study, the highest concentration of organic carbon was observed in the sediments from the coastal sinkhole, likely originating from the surrounding vegetation and from seawater intrusion. We hypothesize that the differences in nutrients found at these two types of sinkholes influence the structure of their microbial communities.

Other environmental factors such as pH and dissolved oxygen (DO), may also contribute significantly to the composition and structure of microbial communities, as seen in freshwater lake sediments^22^. Davis and Garey^20^ reported distinct microbial communities with unique functions for each water layer from an anchialine sinkhole from the Florida karst aquifer and suggested that this occurred as a result of the influence of the hydrochemistry, including differences in the concentration carbon and other nutrients from the environment^20^. Analyses of the sinkhole caves from the Yucatan underwater river support observations that physical and chemical parameters create distinct ecological niches which host unique microbes, as a high abundance of exclusive (not shared) ASVs were observed in the three sediment zones at both locations.

The taxonomic diversity from the coastal and inland sinkholes included Chloroflexi, Crenarchaeota, Desulfobacterota, Proteobacteria, Nitrospirota, Bacteroidota, and Firmicutes as the most abundant phyla in the sediment samples, however, there were differences in the relative abundance associated with the type of sinkhole and sediment zone. Some of these phyla (Chloroflexi, Proteobacteria, and Bacteroidetes) have been reported in sediments from freshwater karst sinkholes from Lake Huron^25^ in water and sediments from other sinkholes in the Yucatan peninsula^6^, and in the karst caves bacteriome from southwest China^21^. A study that included coastal marine sediments from two sites in the Yucatan peninsula, showed high abundances of *Spirochaeta*, *Desulfococcus*, *Clostridium, Psychrobacter*^26^, four genera that were abundant in the coastal sinkhole. However, *Desulfococcus*, *Synechococcus* were also abundant in the inland sinkhole. Of the most abundant genera reported for sediments from different marine environments in the Yucatan coast^23^, *Acinetobacter*, *Desulfotignum*, *Desulfovibrio*, *Pseudomonas*, *Sedimenticola*, and *Sulfurimonas* were also present in the coastal sinkhole while only *Pseudomonas* and *Sedimenticola* were also present in the inland sinkhole^23^. The high number of families shared between the coastal sinkhole and marine sediments from the Yucatan coast, together with the salinity levels registered at the bottom layer of the water column in the coastal sinkhole, suggest an interconnection between these two environments which shapes the microbial communities present in the sediments of caverns and caves of this sinkhole. The genus *Nitrospira* was abundant in the WM from the coastal sinkhole and in all sediment zones from the inland sinkhole. This genus has been reported as one of the most abundant in the surface of speleothems from El Zapote coastal sinkhole^2^, and is considered a complete ammonia oxidizer (comammox), meaning it converts ammonia to nitrate through nitrite. A negative correlation between abundance of this genus and salinity has been reported before, which could explain the low concentration of *Nitrospira* in the cavern and cave from the coastal sinkhole, where the highest salinity was observed^27^. Connectivity between coastal sinkholes and the ocean, as well as the terrestrial input of soil organic matter (OM) has been reported for the underground karst aquifer in the Yucatan peninsula^13^. As in other sediments, degradation of OM is carried out by several MFGs including acetogenic bacteria, methanogens, and sulfate reducers^13,20,28^. When these MFGs were analyzed in coastal and inland sinkholes, differences in their relative abundances were clearly marked by the type of sinkhole and by the sediment zone analyzed, supporting the hypothesis that environmental differences drive microbial community distributions in these niches. The high abundance of sulfate-reducing bacteria (SRB) in the three sediment zones from the coastal sinkhole suggests that sulfate reduction is a predominant function. SRB degrade organic matter using sulfate with sulfide as waste or end-product^19,30^, originating hydrogen sulfide (H_2_S)^29^, which could explain the low concentration of sulfate the hydrogen sulfide (H_2_S) cloud observed and previously reported in the WM zone of El Zapote coastal sinkhole^30^. In this study, high levels of sulfate (SO^−4^) were measured in the water samples from the cavern and cave zones from El Zapote sinkhole, which could be associated with sulfate-rich deposits, such as gypsum beds, which have been reported in other sinkholes from the Yucatan peninsula (up to 2400 mg/L of sulfate concentration)^3^. However, we do not disregard other possible sources of sulfate, associated with seawater intrusion or as a product of sulfide or sulfur oxidation^29,31^ by sulfur-oxidizing bacteria detected in this study.

The inland sinkhole had a low concentration of sulfate and low abundances of SRB. The high abundance of methanogenic bacteria in the WM zone from the coastal sinkhole detected in the MFG analysis supports the previous hypothesis of acetoclastic methanogenesis due to high inputs of organic matter^13^. Methylotrophic bacteria were most abundant at the inland sinkhole in the WM zone, suggesting the presence of methyl compounds, such as methane or methanol which can be used as a source of carbon and energy^32^. High methane concentrations have been quantified in shallow water masses from the Yucatan aquifer system^13^, consistent with observations from this study. ‘*Candidatus Methylomirabilis*’ was identified in the sediment of the WM zone from Noh-Mozón and has been previously described as being able to perform nitrite-dependent anaerobic methane oxidation, using methane as electron donor and nitrate and nitrite as electron acceptors^33^, which would be possible in these sediments considering the low levels of oxygen (average of 2.3 mg/L) detected in the water column above them and assuming this would lead to lower levels of oxygen in the sediments. We hypothesize that bacteria from this genus could be using the nitrite produced by ammonia oxidizing bacteria and archaea observed in this zone (*Nitrosomonadaceae, Nitrospiraceae, and Nitrosococcaceae*). The low abundance of methanotrophic microbes in the coastal sinkhole (mainly the cavern and cave zones) could be derived from the high concentrations of hydrogen sulfide previously reported at this location, which have been suggested to be toxic to methane-oxidizing microbes^34^. Therefore, a decrease in the anaerobic oxidation of methane, and a poor methane removal capacity is hypothesized in the sediments from this coastal sinkhole. Further research could focus on the influence of the saline intrusion on methanotrophic microbes and methane levels in El Zapote sinkhole sediments. As expected, photosynthetic bacterial abundances differed with the type of sinkhole and sediment zone. Both sinkholes are so the presence of daylight can start the photosynthetic process which would occur most in the WM zone. However, only the inland sinkhole showed a high abundance of photosynthetic bacteria within its WM zone. The coastal sinkhole water column and sediments would be deprived of photosynthetic bacteria since the water source is the underground aquifer, lacks photosynthesizers. Ammonia oxidizing bacteria (AOB) and archaea (AOA), and nitrite-oxidizing bacteria (NOB) were relatively abundant in the three sediment zones from the inland sinkhole and in the WM from the coastal sinkhole, these observation at the WM from El Zapote agree with previous observations^2^. Anaerobic ammonium oxidation (anammox) uses nitrite (as a product of nitrate reduction), as electron acceptor^35^. The high levels of nitrate concentration in the water column from the three sediment zones at the inland sinkhole and at the WM from the coastal sinkhole may influence the abundance of AOB, AOA and NOB in the sediments from these zones, while NH_4_^+^ and nitrite values were below detection limit (< 0.2 mg/L and < 1.5 mg/L, respectively) in all sediment zones at both sinkholes, suggesting an oxidation ammonium pathway with N_2_ and 2H_2_O as products^35^, and a rapid cycling of ammonia and nitrite in the sinkhole sediments. Nitrite oxidation could also be responsible for the high NO_3_^−^ concentrations in the water column from the sinkholes, however, we do not discard other sources of NO_3_^−^ including the agricultural and wastewater infiltration into the groundwater aquifer ecosystem^4^, or the presence of abundant bat population which has been previously associated with higher nitrate concentrations in caves from the Yucatan peninsula^8^. Lower concentrations of nitrate (< 1.5 mg/L) in the sediments from caverns and caves correlate with low abundance of NOB. It is thus possible that continuous input of organic matter favors microbial groups associated with the nitrogen cycle (ammonia, nitrite, denitrification) in the sinkholes (mainly the inland sinkhole). Further research could investigate the effects of natural and anthropogenic sources of OM, on the sediment microbial communities in underground caves from the aquifer in the Yucatan peninsula.

When the microbial communities from the two sinkholes were compared to several environments from the Yucatan peninsula, there was a clear difference between communities from water, soil/sediment and speleothems. These differences between the water column and sediment communities have been previously reported in lakes^22^, karst caves^6^, and marine environments^23,24^. We were unable to perform a comparative analysis of environmental variables from sediments and water samples from our study areas since cations, anions, and in situ parameters were not measured in sediments, and TOC, TIC, and total N were only measured in the water column. However, comparisons to other environments suggest that the water and sediments from sinkholes are unique environments which can provide specific ecological niches and host distinctive microbes with specialized functions.

In this study, we successfully revealed differences in the sediment microbial community structure associated with physical and chemical characteristics within two types of sinkholes (coastal and inland) and within defined sediment zones (WM, cavern, and cave). Based on the analyses of environmental variables, taxonomic diversity, alpha and beta diversity, and the potential metabolic function of these microbial communities, we demonstrate that each sinkhole represents a unique environment harboring different microbial assemblages. Therefore, we plan to pursue future research that includes investigating a greater number of coastal and inland sinkholes to determine if the patterns found in this study apply to the majority of coastal and inland sinkholes in the Yucatan peninsula. We recognize the limitations of partial sequencing of the 16S rRNA gene and, therefore, all hypotheses about MFGs environmental functions should investigated by further experimental and metagenomic studies that shed more light on their actual roles in geochemical cycles occurring in the underground aquifer of the Yucatan peninsula.

## Methodology

### Study sites, sample collection and environmental determinations

Water and sediment samples were collected from the underground karst aquifer at a coastal (El Zapote) and an inland (Noh-Mozón) sinkhole in the Yucatan peninsula in August and November 2019, respectively (Fig. 1). The sinkhole El Zapote is located at Puerto Morelos, Quintana Roo, Mexico (20°51′27.78″N 87°07′35.93″ W). It is a bottle-shaped cave with a stratified water column of freshwater that reaches approximately 35 m depth, followed by an H_2_S- enriched layer redoxcline at ~ 35.2 m, a halocline (~ 36.6 m), and a saltwater layer (~ 38 m) that reaches 54 m depth (Fig. 1). The Noh-Mozón sinkhole is located in a remote area of the NW Yucatan peninsula surrounded by shrubs and agricultural fields, in the community of Pixyah, Yucatán, Mexico (20°37′23.9 N, − 89°23′03.1′W). The sinkhole is semi-open, the water column is clear, and entirely composed of freshwater. The sinkhole has a 9 m free fall from ground level to the water, with a maximum depth of 45 m. Its current uses are related to tourism activities, such as swimming, diving, camping, and nature-watching (Fig. 1). For both sinkholes, water and sediment samples were collected in three sediment zones. The first zone (the water mirror, WM) was determined to be the shallowest zone, completely exposed to solar radiation and between 0 and 39 m in the coastal sinkhole, and between 0 and 15 m in the inland sinkhole. The second zone (the cavern) was defined as the zone with low solar radiation and between 39 and 51 m in the coastal, and 15 and 20 m in the inland sinkhole. Finally, the cave was defined as the zone with null solar radiation and depths between 51 and 54 m, and 20 and 34 m in the coastal and inland sinkholes, respectively. Six points were sampled per sediment zone as a transect from the WM to the cave, and 3 biological sediment replicates were collected per point. An extra sampling point (in triplicate) was also taken from outside the sinkhole, bulk soil located ~ 10 m away and opposite from the main entrance to the sinkhole as a control. The samples were collected in 50 ml sterile Falcon tubes (~ 50 g per tube) and transported to the laboratory at 4 °C, a portion of the sample was frozen and processed to determine organic and inorganic carbon, total nitrogen, and total phosphorus at the Soils and Plants Analysis Laboratory, “El Colegio de la Frontera Sur” (ECOSUR). RNAlater (Sigma) was added to the rest of the sediment samples and frozen at − 80 °C until further processing. Water samples (500 mL) were collected from each sediment zone using Nalgene bottles and were stored at 4 °C for further nutrient analysis. In situ parameters such as temperature (°C), salinity (psu), dissolved oxygen (mg/L), electrical conductivity (ms/cm), and total dissolved solids (TDS, mg/L) were determined with a multiparameter water probe EXO1 (Xylem Analytics, Norway) for the coastal sinkhole and a YSI Professional Plus handheld multiparameter for the inland sinkhole. Cations (Na^+^, K^+^, Ca^2+^, Mg^2+^) and anions (HCO_3_^−^, Cl^−^, NO_3_^−^, SO_4_^2−^) were measured by anion exchange chromatography at the Geochemical and Mineralogy Laboratory, Institute of Geology, UNAM.

### Statistical analysis for environmental variables

A Shapiro–Wilk normality test was performed to identify data distribution followed by a Kruskal–Wallis test to find out if environmental variables from the coastal sinkhole differed significantly by “sediment zone”, both tests were conducted using Rstudio^36^. Principal component analysis (PCA) of normalized data using a Euclidean distance matrix was performed to explore and visualize distribution similarities between the water (HCO_3_^−^, Cl^−^, NO_3_^−^, SO_4_^2−^, Na^+^, K^+^, Ca^2+^, Mg^2+^) and sediment (P total, C organic, C inorganic, total N) environmental variables from coastal and inland sinkholes (Table S1) using the R package ‘‘vegan’’ (v1.17–2)^37^.

### DNA extraction, 16S rRNA library preparation and sequencing

Approximately 250 mg of sediment from each sample was used for isolation of the environmental DNA using the QIAGEN PowerSoil kit following the manufacturer’s instructions. The amplification of the V4 region of the 16S rRNA was performed in triplicate (n = 9 per sampling point) using the primers 515F (5′-GTGCCAGCMGCCGCGGTA A-3′) and 806R (5′-GGACTACHVGGGTWTCTAAT3′) following the thermal conditions reported by Caporaso et al.^38^. PCR products were quantified using a Qubit Fluorometer (Promega) and 20 ng/sample were sent to the Yale Center for Genome Analysis (CT, USA) for sequencing with an Illumina MiSeq instrument (250-bp paired-end reads).

### Sequence analyses

Demultiplexed sequences were imported into the Quantitative Insights Into Microbial Ecology 2 program (QIIME 2 ver. 2018.8.0)^39^ using the “tools import” plugin. The Divisive Amplicon Denoising Algorithm 2 (DADA2)^40^ was used to quality filter, trim, denoise, and merge pairs of reads. Chimeric sequences were removed using the consensus method. Amplicon sequence variants (ASVs) were taxonomically assigned using the feature-classifier^2^ plugin implemented in QIIME2 against the SILVA SSU non-redundant database (138 release). To increase taxonomic resolution, sequences that were not taxonomically classified at the genus level were blasted against the NCBI database and their nearest neighbor, based on sequence identity, was selected. ASVs assigned to chloroplast, mitochondria, and sequences with less than ten counts in at least one sample, and identified in less than five sediment samples, were removed.

### Microbial composition and community structure analysis

The filtered ASVs were normalized based on the number of sequences from the library with the lowest sequencing depth within each comparison group. Three types of normalized ASV data were performed to test the importance of the type of sinkhole and sediment zone in the microbial community structure of sediment samples from underground karst aquifer at the coastal site: (a) the data group 1 defined as those ASVs present in the 104 sediment samples from coastal and inland sinkholes, which were normalized to 8204 reads per sample, (b) data group 2 defined as those ASVs present in 51 sediment samples from the coastal sinkhole with a rarefaction of 21,432 reads per sample, and (c) data group 3 defined as those ASVs present in the 53 sediment samples from the inland sinkhole, which were rarefied to 8204 reads per sample. Rarefaction curves were computed directly using QIIME2 for the three data groups^39^. The soil samples (soil outside of the sinkholes) were used as a control group with their respective normalization for each data group mentioned above.

The Rea pipeline^41^ was used to estimate the ASV richness of data group 1, and to test statistical differences among group comparisons a Kruskal–Wallis test was used. Heatmaps were built to visualize the 20 most abundant phyla found in the data group 1, and for the 20 most abundant genera from data group 2 and 3, using the R package gplots v.3.0.1^42^. To measure and visualize the differences in the microbial community among sediment samples (beta-diversity) a non-metric multidimensional scaling (nMDS) ordination with a Bray–Curtis distance analysis^43^ was performed for the three data groups using the R package ‘‘vegan’’^37^. In addition, for data groups 2 and 3 a nMDs with a weighted Unifrac distance analysis was used. Differences between all groups and pairwise communities were calculated with a permutational multivariate analysis of variance (PERMANOVA) in VEGAN Package^37^. To visualize shared ASVs and genera between the different sediment zones in the data groups 2 and 3, we constructed Venn diagrams using the R package VennDiagram v.1.6.20^44^. The potential metabolic function of the microbial communities (at Family and Genus level) from coastal (data group 2) and inland sinkholes (data group 3) was assigned by a review of the literature of these taxonomic levels and their metabolic function capacity, and defined as microbial functional group (MFG).

A barplot was built to visualize these MFG using Rstudio^36^. ‘Heat trees’ of taxonomic diversity from the sediment samples (data group 2 and 3) were developed using the R package ‘metacodeR’ v.0.2.1^45^. In these plots, the node width and color indicate the number of reads assigned to each taxon, and the 20 most abundant in each taxonomic level were highlighted (Phylum to Genus level). A comparative analysis of published data with other studies in the Yucatan peninsula (sinkholes and marine environments, Supplementary Table S5) using 16S rRNA amplicons was performed considering a rarefaction to 3490 reads per sample. An nMDS with Bray–Curtis distance analysis was created using VEGAN package in R^37^ and used to visualize the similarities between samples of the different studies^2,13,26^. To visualize families shared among the six different environment an Upsetplot was constructed using R package UpsetR^46^.

## Supplementary Information

Supplementary Information.

## Data Availability

Raw reads were subsequently deposited into the National Center for Biotechnology Information (NCBI) Sequence Read Archive (SRA) database (PRJNA721655).
