# Supplementary material for: Changes in the sediment microbial community structure of coastal and inland sinkholes of a karst ecosystem from the Yucatan peninsula
Source: Sci Rep. 2022 Jan 21;12:1110. doi: 10.1038/s41598-022-05135-9 (PMC8782880; doi:10.1038/s41598-022-05135-9)
Supplement: Supplementary file 1 — Supplementary Information. [file 41598_2022_5135_MOESM1_ESM.pdf]

Changes in the sediment microbial community structure of coastal and inland sinkholes of a karst ecosystem from the Yucatan Peninsula

Pablo Suárez-Moo, Claudia A. Remes-Rodríguez, Norma A. Márquez -Velázquez, Luisa I. Falcon, José Q. García-Maldonado, Alejandra Prieto-Davó.

Supplementary Figure 1. *In situ* environmental variables measured in the water samples from the coastal and inland sinkholes

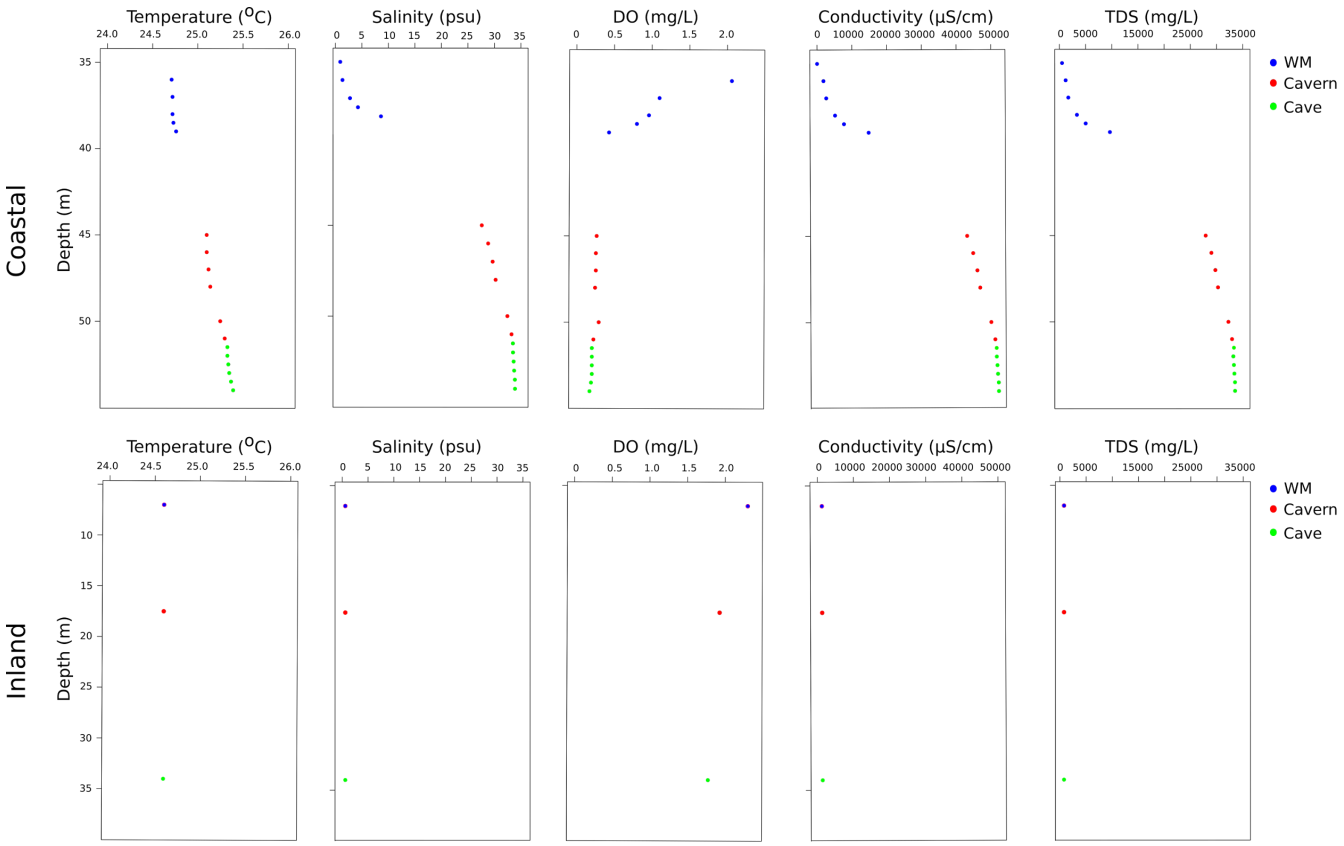

Supplementary Figure 2. Principal component analysis (PCA) based on the environmental variables from water and sediments for each sampled zone in both sinkholes.

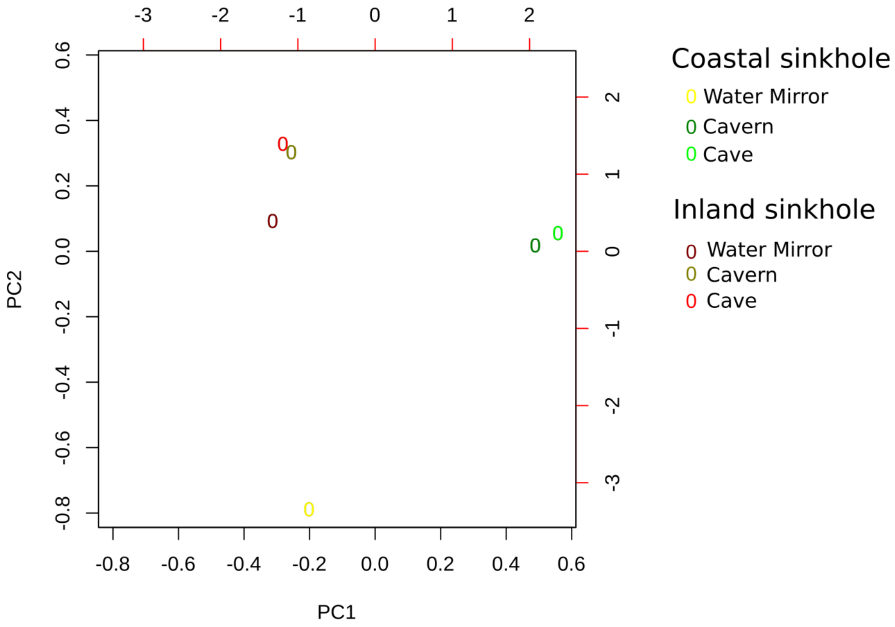

Supplementary Figure 3. Rarefaction curves of the observed ASVs in the sediment samples from the coastal and inland sinkholes. A) data group 1 (110 samples/8204 reads by sample) B) data group 2 (Coastal sinkhole, 51 samples/21432 reads by sample) C) data group 3 (Inland sinkhole, 53 samples/8204 reads by sample). WM, water mirror; CR, cavern; CV, cave; OS, outside.

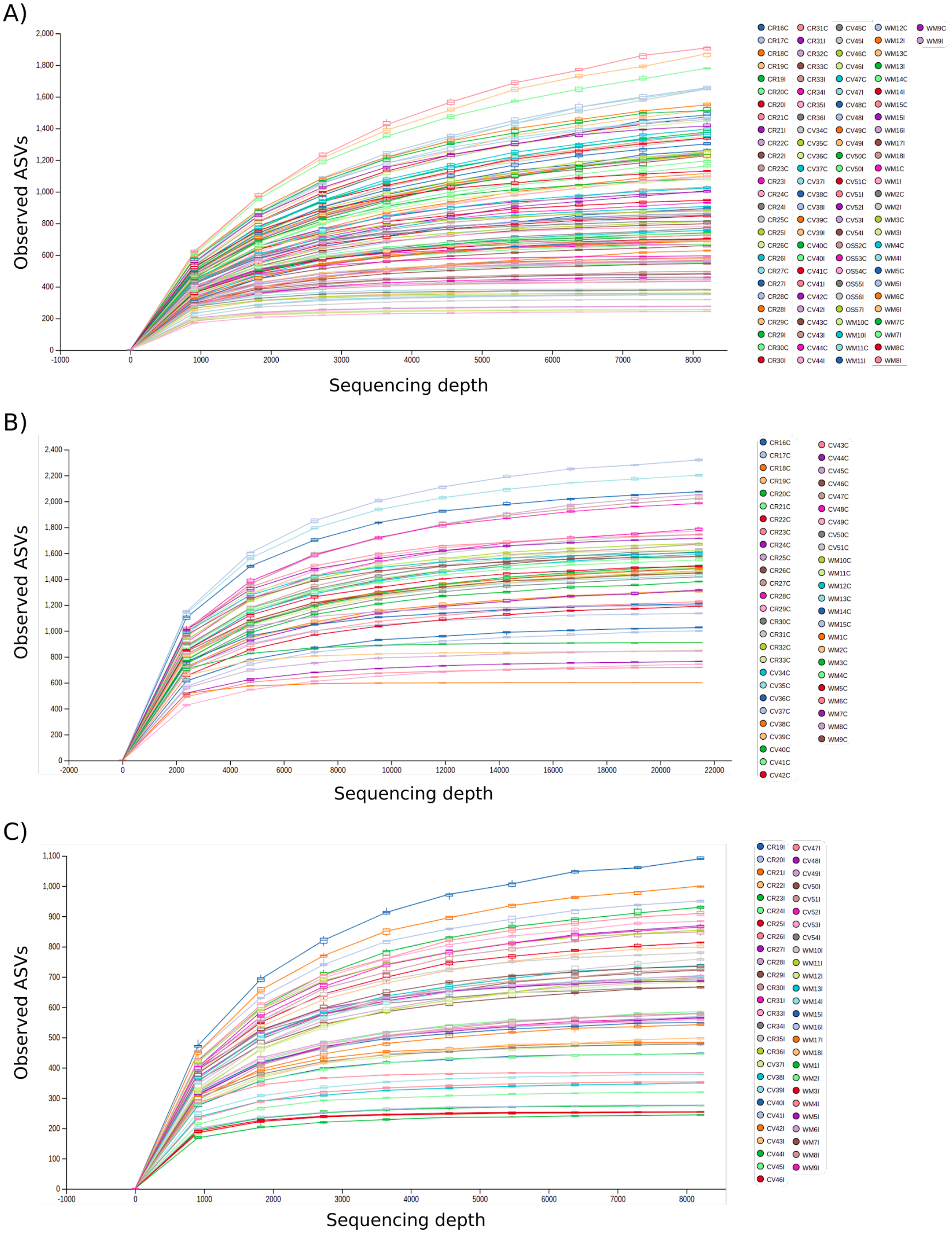

Supplementary Figure 4. Taxonomic diversity and abundance of Bacteria and Archaea found in the sediment zones from the coastal (A) and inland sinkholes (B). The node width and color indicate the number of reads assigned to each taxon. The 20 most abundant taxonomic levels (from genus to phylum) are described.

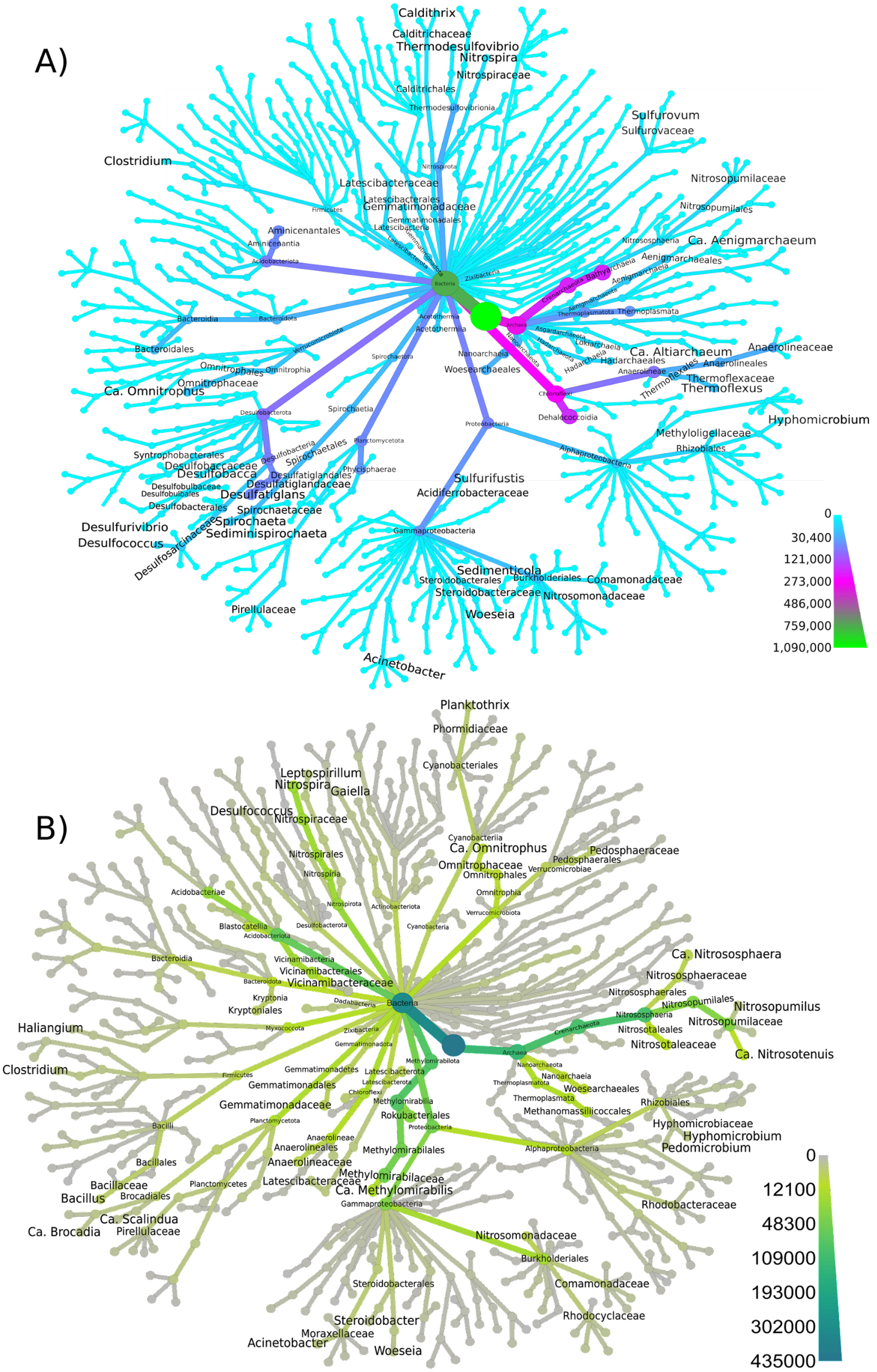

Supplementary Figure 5. Venn diagram showing the shared ASVs (in black) and identified genera (in blue) between the sediment zones and the bulk soil control in the coastal (A) and inland (B) sinkholes. The percentage is shown in parenthesis.

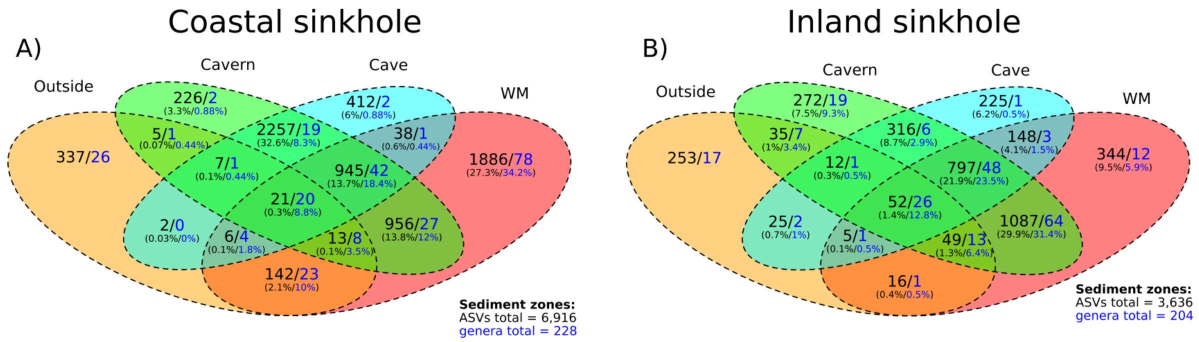

Supplementary Figure 6. Beta diversity analysis of sediment samples from coastal (A) and inland (B) sinkholes. Non-metric multidimensional scaling (NMDS) plot based on weighted unifrac distance of the ASVs.

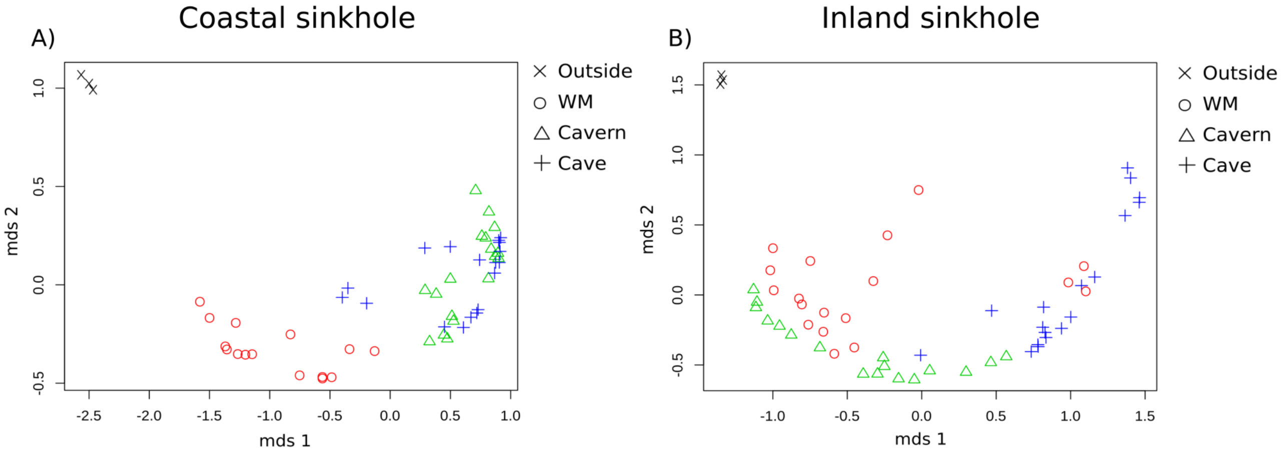

Supplementary Figure 7. Shared and exclusive families among the microbial communities of different environments from the Yucatan peninsula.

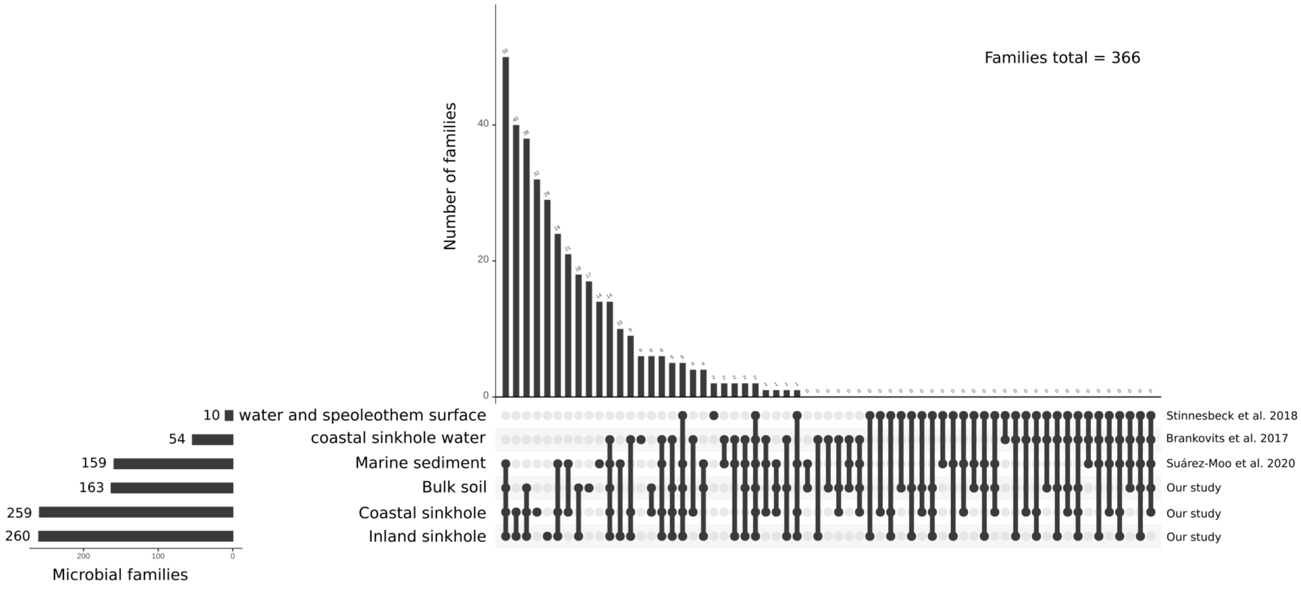

Supplementary Table S1. Physicochemical composition of water and sediment samples from coastal and inland sinkholes

		Water samples (mg/L)								Sediment samples (%)		
Site	Zone	HCO_3^-	Cl-	NO_3^-	SO_4^{-2}	Na+	K+	Ca^{2+}	Mg^{2+}	C organic	C inorganic	N_2 total
Coastal	WM	379.5	255.1	4.2	34.7	130	5.8	73.5	32.6	3.9	11.8	0.58
Coastal	Cavern	200.9	12770	<LD	1858.7	6474.4	249.8	384.5	791	7.2	12.48	0.77
Coastal	Cave	245.6	14957	<LD	2027.7	7585.7	291.4	452.3	932	7.55	12.18	0.7
Inland	WM	200.9	601.4	5.3	83.9	313.5	10.9	47.6	45.6	0.35	11.97	0.12
Inland	Cavern	141.4	207.5	4.2	30.2	108.5	3.9	29.6	21.6	<LD	14.31	0.08
Inland	Cave	111.6	25.3	1.4	6	14.9	0.6	22.8	8.3	0.52	10.48	0.19

Supplementary Table S2. Statistical differences after Kruskal–Wallis test of *in situ* water variables between sampled zones in the coastal sinkhole

	Shapiro test		Kruskal-Wallis test	
**Environmental variable**	**W**	**P-value**	**chi-squared**	**P-value**
**Depth (m)**	0.86847	0.02078	15.174	0.001
**ODO (mg/L)**	0.63196	2.16E-05	15.348	0.001
**Temperature (oC)**	0.79989	0.002022	13.274	0.001
**Cond (µS/cm)**	0.70396	0.0001282	15.174	0.001
**Salinity (psu)**	0.70479	0.0001311	15.174	0.001
**TDS (mg/L)**	0.40069	2.07E-07	15.174	0.001

Supplementary Table S3. Raw read information, number of high-quality reads and ASVs obtained from each sediment zone of the coastal and inland sinkholes

				High-quality reads		High-quality reads with normalization*					
			Raw data	Non normalization		Data group 1		Data group 2		Data group 3	
**Site**	**Zone**	**#samples**	**# reads**	**# reads**	**# ASVs**	**# reads**	**# ASVs**	**# reads**	**# ASVs**	**# reads**	**# ASVs**
Coastal	WM	15	2290824	1070418	4045	123060	3905	321480	4007	x	x
Coastal	Cavern	18	3919739	2424930	4542	147672	4157	385776	4430	x	x
Coastal	Cave	18	3619791	1980466	3804	147672	3501	385776	3688	x	x
Inland	WM	18	1455337	545828	2564	147672	2502	x	x	147672	2498
Inland	Cavern	17	1752541	660953	2665	139468	2621	x	x	139468	2620
Inland	Cave	18	1188980	542638	1612	147672	1570	x	x	147672	1580
Total			14227212	7225233	9422	853216	9402	1093032	6916	434812	3383

*Rarefaction: 8204 reads per sample in group 1; 21432 reads per sample in group 2; 8204 per sample in group 3

Supplementary Table S4. Detailed information for sediment samples of the coastal and inland sinkholes. The number of high-quality reads and ASVs obtained for each sample are also shown.

ID_sample	Site	Zone	#Reads	#ASVs	ID_sample	Site	Zone	#Reads	#ASVs
OS52Z	Coastal	Outside	56645	446	OS55N	Inland	Outside	33847	384
OS53Z	Coastal	Outside	67304	480	OS56N	Inland	Outside	23561	351
OS54Z	Coastal	Outside	52855	451	OS57N	Inland	Outside	25499	361
WM10Z	Coastal	WM	93050	1738	WM10N	Inland	WM	56306	835
WM11Z	Coastal	WM	57392	1653	WM11N	Inland	WM	42502	752
WM12Z	Coastal	WM	58099	1629	WM12N	Inland	WM	35929	727
WM13Z	Coastal	WM	104783	2300	WM13N	Inland	WM	50423	792
WM14Z	Coastal	WM	96258	2158	WM14N	Inland	WM	34468	737
WM15Z	Coastal	WM	137457	2478	WM15N	Inland	WM	19482	453
WM1Z	Coastal	WM	21432	600	WM16N	Inland	WM	13173	278
WM2Z	Coastal	WM	32503	843	WM17N	Inland	WM	42987	582
WM3Z	Coastal	WM	34884	910	WM18N	Inland	WM	43838	522
WM4Z	Coastal	WM	76166	1628	WM1N	Inland	WM	19726	246
WM5Z	Coastal	WM	76872	1550	WM2N	Inland	WM	19383	454
WM6Z	Coastal	WM	70852	1784	WM3N	Inland	WM	11732	255
WM7Z	Coastal	WM	91587	1764	WM4N	Inland	WM	8204	384
WM8Z	Coastal	WM	51744	1205	WM5N	Inland	WM	21743	699
WM9Z	Coastal	WM	67339	1602	WM6N	Inland	WM	24356	711
CR16Z	Coastal	Cavern	118419	1085	WM7N	Inland	WM	27730	760
CR17Z	Coastal	Cavern	101143	1064	WM8N	Inland	WM	32960	613
CR18Z	Coastal	Cavern	100412	1385	WM9N	Inland	WM	40886	920
CR19Z	Coastal	Cavern	146354	1416	CR19N	Inland	Cavern	45281	1167
CR20Z	Coastal	Cavern	112879	1466	CR20N	Inland	Cavern	37789	1008
CR21Z	Coastal	Cavern	131512	1601	CR21N	Inland	Cavern	37851	1055
CR22Z	Coastal	Cavern	155081	1602	CR22N	Inland	Cavern	39972	855
CR23Z	Coastal	Cavern	153075	1341	CR23N	Inland	Cavern	44115	1000
CR24Z	Coastal	Cavern	158401	1441	CR24N	Inland	Cavern	34308	610
CR25Z	Coastal	Cavern	210852	2281	CR25N	Inland	Cavern	40058	862
CR26Z	Coastal	Cavern	128182	1720	CR26N	Inland	Cavern	50662	978
CR27Z	Coastal	Cavern	156128	2202	CR27N	Inland	Cavern	45272	927
CR28Z	Coastal	Cavern	125446	2119	CR28N	Inland	Cavern	45557	740
CR29Z	Coastal	Cavern	97773	1855	CR29N	Inland	Cavern	46692	784
CR30Z	Coastal	Cavern	103156	1515	CR30N	Inland	Cavern	41203	907
CR31Z	Coastal	Cavern	169168	1848	CR31N	Inland	Cavern	34996	589
CR32Z	Coastal	Cavern	126861	1571	CR33N	Inland	Cavern	38436	938
CR33Z	Coastal	Cavern	130088	1551	CR34N	Inland	Cavern	20327	679
CV34Z	Coastal	Cave	157530	1717	CR35N	Inland	Cavern	26111	806
CV35Z	Coastal	Cave	113229	1513	CR36N	Inland	Cavern	32323	891
CV36Z	Coastal	Cave	78434	1247	CV37N	Inland	Cave	31569	495
CV37Z	Coastal	Cave	62857	1163	CV38N	Inland	Cave	26122	359
CV38Z	Coastal	Cave	119608	1581	CV39N	Inland	Cave	27716	386
CV39Z	Coastal	Cave	127060	1546	CV40N	Inland	Cave	40310	570
CV40Z	Coastal	Cave	136298	1628	CV41N	Inland	Cave	54384	602
CV41Z	Coastal	Cave	114428	1644	CV42N	Inland	Cave	24658	494
CV42Z	Coastal	Cave	130044	1278	CV43N	Inland	Cave	18879	257
CV43Z	Coastal	Cave	64452	731	CV44N	Inland	Cave	19689	277
CV44Z	Coastal	Cave	75069	782	CV45N	Inland	Cave	16939	321
CV45Z	Coastal	Cave	74196	868	CV46N	Inland	Cave	17160	255
CV46Z	Coastal	Cave	96128	1511	CV47N	Inland	Cave	24584	360
CV47Z	Coastal	Cave	130401	1781	CV48N	Inland	Cave	30852	585
CV48Z	Coastal	Cave	157210	1926	CV49N	Inland	Cave	42296	602
CV49Z	Coastal	Cave	97039	789	CV50N	Inland	Cave	37686	694
CV50Z	Coastal	Cave	126592	1732	CV51N	Inland	Cave	45747	771
CV51Z	Coastal	Cave	119891	1857	CV52N	Inland	Cave	37639	736
					CV53N	Inland	Cave	24682	572
					CV54N	Inland	Cave	21726	488

Supplementary Table S5. Microbial community studies used in the comparative analysis

Type of sample	Enviroment	Site	#Libraries	16S region	Primers	NCBI Accesion number	Autor
Water	Sinkhole	Yucatan peninsula	16	V4	515F and 806R	SRP109857	Brankovits et al. 2017
Sediment	Marine coast	Yucatan peninsula	12	V4	515F and 806R	PRJNA631553	Suárez-Moo et al. 2020
Water and speleothem surface	Sinkhole	Yucatan peninsula	4	V3 and V4	Bact_341F and Bact_805R<br>A519F and U906R	PRJNA389527	Stinnesbeck et al. 2018
Sediment	Sinkhole	Yucatan peninsula	110	V4	515F and 806R	XXX	Our study
